# Semen evaluation: methodological advancements in sperm quality-specific fertility assessment — A review

**DOI:** 10.5713/ab.21.0072

**Published:** 2021-04-23

**Authors:** Bereket Molla Tanga, Ahmad Yar Qamar, Sanan Raza, Seonggyu Bang, Xun Fang, Kiyoung Yoon, Jongki Cho

**Affiliations:** 1 College of Veterinary Medicine, Chungnam National University, Daejeon 34134, Korea; 2 Faculty of Veterinary Medicine, Hawassa University, 05, Hawassa, Ethiopia; 3 Department of Clinical Sciences, College of Veterinary and Animal Sciences, Jhang 35200, Sub-campus University of Veterinary and Animal Sciences, Lahore 54000, Pakistan; 4 Department of Reproduction and Artificial Insemination, Faculty of Veterinary Medicine, Aydin Adnan Menderes University, Aydin 09016, Turkey; 5 Department of Companion Animal, Shingu College, Seongnam 13174, Korea

**Keywords:** Biomarkers, Fertility, Sperm Biology, Sperm Evaluation, Computer-assisted Sperm Analyzers (CASA)

## Abstract

Assessment of male fertility is based on the evaluation of sperm. Semen evaluation measures various sperm quality parameters as fertility indicators. However, semen evaluation has limitations, and it requires the advancement and application of strict quality control methods to interpret the results. This article reviews the recent advances in evaluating various sperm-specific quality characteristics and methodologies, with the help of different assays to assess sperm-fertility status. Sperm evaluation methods that include conventional microscopic methods, computer-assisted sperm analyzers (CASA), and flow cytometric analysis, provide precise information related to sperm morphology and function. Moreover, profiling fertility-related biomarkers in sperm or seminal plasma can be helpful in predicting fertility. Identification of different sperm proteins and diagnosis of DNA damage has positively contributed to the existing pool of knowledge about sperm physiology and molecular anomalies associated with different infertility issues in males. Advances in methods and sperm-specific evaluation has subsequently resulted in a better understanding of sperm biology that has improved the diagnosis and clinical management of male factor infertility. Accurate sperm evaluation is of paramount importance in the application of artificial insemination and assisted reproductive technology. However, no single test can precisely determine fertility; the selection of an appropriate test or a set of tests and parameters is required to accurately determine the fertility of specific animal species. Therefore, a need to further calibrate the CASA and advance the gene expression tests is recommended for faster and field-level applications.

## INTRODUCTION

Fertility defines the rate of reproduction and ability to propagate generations, though it is declining globally for the last two centuries [1]. Fertility rate measures the number of offspring that can be produced during the life span, and fecundity measures the biological potential of the number of offspring that can be obtained during a lifetime [2]. In either case, it is determined by the proper functioning of the reproductive system, its anatomy and physiology of organs, glands, ducts, and testicles/ovaries. In male animals, fertility can be evaluated from the number of sperm produced with the capability to fertilize the oocyte both in *in-vivo* and *in-vitro* and is measured with the help of semen analysis, even though it has limitations [3]. Semen analysis is advanced, but the reliability and validity of fertility determination in humans [4] and various species of animals [5] are widely controversial in the literature.

Semen primarily consists of two main components: the sperm and seminal fluids; sperm are produced in the seminiferous tubules of the testicles, and the seminal fluids, in the accessory sex glands and excurrent ducts. Both of these components are examined in semen analysis in terms of sperm count and semen volume [6]. Semen analysis provides information about normal operations of the testicular machinery for the production of sperm, and the fluid part. Furthermore, it evaluates the sperm to assess fertility [7] or the success of surgical procedures such as vasectomy [8]. Semen analysis includes examining the physical characteristics of semen (color, odor, pH, viscosity, and liquefaction), volume, concentration, morphology, sperm motility and progression, conducted repeatedly at different intervals [9]. In domestic animals, fertility determination is very important, particularly in male animals used for breeding purposes and artificial insemination (AI). The infertility issues associated with sperm can adversely affect the breeding outcomes of large herds of animals [10]. Therefore, the assessment of sperm fertility in breeding bulls is of paramount importance [11–13].

For a long time, semen evaluation was believed to be the single most important laboratory test for assessing male fertility [14] however, it still remains complex and difficult to standardize. Several confounders have made the goal of one-test evaluation quite difficult to accomplish, such as the inability to strictly control quality to obtain meaningful results, variability among laboratories, and inability to comply with standard procedures to accomplish correct interpretations from results [15]. The structural evaluation of sperm is based on its appearance, morphology, concentration, plasma membrane integrity, and chromatin integrity, whereas functional evaluation is based on its motility, capacitation, and acrosomal reaction [16]. Advances in technologies enable the assessment of sperm structure and function, such as plasma membrane and genomic constituents, and enables better determination of sperm fertility potential.

The structure of sperm is peculiar, and functions to transfer the male gametes and required genetic information [17], equipped with a strong flagellum to propel but devoid of cytoplasmic organelles like ribosomes, Golgi apparatus or endoplasmic reticulum [18]. However, sperm contain more of mitochondria, placed strategically, bases of energy source for motility [19]. Structurally, even if consisted of single cell membrane, it has two morphologically and functionally distinct, tail to motion and head containing the genetic material [20]. Different from other cells, DNA is tightly packed, and its integrity is basis for attaining the required fertility [21]. The head has anterior end of the nuclear envelope, referred as acrosome, contains hydrolytic enzymes that help the sperm to penetrate the egg's outer coat by an acrosome reaction [22].

In bulls, semen quantity, quality, and/or health status are responsible for a significant percentage of reproductive failure in cattle production [23]. As AI is widely used in cattle, semen evaluation plays an imperative role not only in the successful establishment of pregnancy but also in facilitating genetic improvement [24], attaining breeding and milk, and meeting production objectives [25]. In bulls, factors including age, breed, time and interval of collection, and season of the year affect the semen quality [26]. Computer-assisted sperm analyzers (CASA) is believed to be a better method for assessing the quality of fresh semen, not only as a faster measure but also in terms of the ability to measure multiple dimensions of sperm fertility, precisely and accurately [27,28]. The criteria for selecting bovine semen samples for preservation or breeding purposes rely on concentration (sperm/mL), initial motility (%), and total normal morphology of sperm (%), but the recommended criteria and points vary [13,25]. There is no doubt that more stringent criteria are required for the optimal evaluation of semen.

In domestic animals, fertility indicators are affected by several factors, including the host environment and nutritional factors; however, in this review, we have focused solely on semen evaluation methods. Semen evaluation can be conducted at different levels, that is semen and sperm, or by assessing the mechanical, physical, or functional characteristics of sperm [29], which directly or indirectly evaluates the sperm-specific quality in terms of attaining fertility. For the convenience of this review, we start with the fertility-related parameters of sperm and subsequently review the different methods and their advancements. Moreover, we describe the measurement values and advantages and disadvantages of specific methods in various species of domestic animals. Semen quality varies depending on animal characteristics, countries, and sometimes farms [30,31].

This article reviews the developments in current semen analysis methods and the knowledge obtained from determining the fertility of sperm. Therefore, we aimed to review the progress in semen evaluation techniques and to make conclusive remarks regarding recent advances in semen evaluation of domestic animals. This review focuses on the reliability and limitations of semen analysis for the diagnosis of fertility.

## SPERM CONCENTRATION AND MORPHOLOGY

Determination of sperm concentration, together with the assessment of motility and morphology, is an important method to determine fertility. Sperm concentration per mL of semen can be determined by counting the sperm in the chamber of a hemocytometer, a microcell, or a photometer. However, there are possibilities of underestimation and overestimation of sperm numbers depending on the types of products used [32]. The calibration of devices to measure concentration is critical to ensure accurate sperm number per dose and produce maximum doses per ejaculate. In addition to these methods, sperm concentration can also be determined with the help of CASA [33], flow cytometer [34], and NucleoCounter SP-100 [35]. CASA can also be used for instant quantification of sperm concentration and motility. The NucleoCounter SP-100 can assess sperm concentration and membrane integrity. NucleoCounter SP-100 is more efficient than the hemocytometer because it is quicker, simpler, objective, and precise. Moreover, it is cost-effective and more user-friendly compared to flow cytometry [35].

A study comparing various methods used for the determination of sperm concentration revealed that flow cytometry was the most precise method [36]. However, before performing flow cytometry, there is a requirement for preliminary assessment of sperm count using a different method to ensure proper semen dilution (close to 250,000 sperms/mL). The spectrophotometer was found to be the second-best option for the precise determination of sperm concentration. However, it might not be an appropriate method for the assessment of sperm samples with low concentrations and volume [36]. Standardizing a laboratory procedure for assessing sperm concentration is affected by factors, which include animal species, the sample size needed, operational frequency, the number of samples assessed per day, and the operational cost.

Morphometric characteristics of sperm are one of the most important indicators of fertility. It is universally accepted that sperm with normal morphology have a significant effect on fertility both *in vivo* and *in vitro*, and it is an integral part of sperm functional test [37–40]. Abnormal types are categorized according to the sperm morphology, including defective head, neck, spacer, and default queue [41]. Different fixation and smear preparation methods are adopted for the assessment of sperm morphology. However, no optimal method has been standardized for any particular animal species. High variability exists within and between laboratories regarding the accurate assessment of sperm morphology. The computerized analysis of sperm morphology is known as automated sperm morphometry analysis (ASMA). ASMA enables morphological assessments of live sperm [42]. This system can efficiently classify normal and abnormal sperm by overcoming technical variations. Moreover, it provides objective estimation, with improved accuracy and precision of sperm morphology assay. Sperm morphology can be better elaborated if staining is applied.

## SPERM MOTILITY

The advancements in sperm motility assessment include the use of the light microscope and phase-contrast microscope, using 20 and 40× objectives, which are reported to yield substantially good results [43]. The microscope should be equipped with a stage warmer that can be adjusted to 37°C and magnification levels should allow clear visualization of the sperm samples [44]. It is advised to avoid the use of a light microscope because of clear visualization issues, as immotile sperm are difficult to identify, especially at low magnification [44]. The inability to identify immotile sperm may result in false high-motility values.

### Motility by phase-contrast microscopy

Phase-contrast microscope is generally used to determine mass motility or progressive sperm motility [45]. Mass motility is a very important parameter for assessing sperm fertility in humans and domestic animals [46–50]. Based on the motility pattern, each sperm is categorized either as possessing progressive motility (PR) or non-progressive motility [51]. While reporting sperm motility, it is necessary to consider total motility (PR+NP) or progressive motility. In terms of fertility, only the percentage of progressively motile sperm was found to be associated with pregnancy rates. Thus, differentiating PR and NP is important [52].

PR is usually expressed as a percentage of sperm motility, which is suggestive of sperm fertility, indicating proper spermatogenesis and maturation of sperm during epididymal transit [53]. In humans, the PR of sperm is mainly affected by thiols of flagellar proteins such as outer dense fiber protein 1, which are oxidized to form disulfides during epididymal transit and the sperm attains motility [53]. In animals, with external fertilization, usually, the exposure of sperm to the fertilization medium results in hypotonic shock that induces activation of motile function [54,55]. In mammals, cAMP signaling pathways and adenylyl cyclase are associated with progressive sperm motility [56,57]. The PR of human sperm is associated with DNA damage and fragmentation, which is vital for successful fertilization [58].

Various factors can affect the results of phase-contrast microscopy, such as, magnification, working conditions (heated stage, ambient temperature), personal experience, and environmental factors [44,59]. Some authors have described specific sperm motility characteristics such as swirling oscillation in warm slide microscopy to measure the mass motility to predict fertility [48].

### Motility by computer-assisted sperm analysis

The CASA is the first major system to analyze motion characteristics of sperm. It provides instant quantification of sperm motility and concentration. CASA is relatively better than phase-contrast microscopy as it can generate accurate and highly reproducible data of different kinetic parameters of sperm [60,61]. Furthermore, CASA largely reduces the subjectivity by overcoming the inherent variability of routine microscopic semen examination [27]. It simply involves the grading of sperm motility into four orders, which are, highly progressively motile, progressively motile, non-progressively motile, and immotile (grade a, b, c, and d). While this presented breakthroughs for sperm-motility quantification, they were largely black boxes with little true verification and skepticism.

In domestic animals, commercial use of a fully automated CASA system was introduced in 1985 [62]. In the beginning, the CASA systems utilized for sperm analysis were based on an expert vision system developed for other purposes [63,64]. Later on, in 1992, old systems were replaced with automated CASA systems specifically developed for determining sperm-morphology of bull [65], dog [66], human [67], and stallion [68]. Many CASA systems utilize different filters to better recognize sperm and represent great improvements. For streamline accuracy, intelligent filters form part of the software that can detect if a “particle” has a tail. This became an advantage in case of contaminated semen samples which are difficult to analyze using other software.

CASA generated motility data of sperm, (percentage and concentration of motile sperm) which is very helpful for the prediction of fertility in humans *in vivo* [69] and *in vitro* [70]. The development of CASA has enabled the evaluation of various fertility parameters. The recent development of CASA technology to analyze key kinematic functionality parameters such as hyperactivation, enabling visualization of sperm in three dimensions, and measuring the flagellar and sperm tracking (FAST) in a quantitative way will be of paramount importance to assess sperm characteristics and fertility in detail [62,71].

However, the CASA system has some limitations, affected by the higher sperm concentration, which interferes with progressive motility [37,72,73]. During semen analysis, shortcomings such as low contrast images and artifacts of dirt can negatively affect the accuracy of the CASA software. In such cases, sperm concentration is greatly overestimated and the percentage motility of sperm is underestimated. These issues remained inherent in most CASA systems only to be partly resolved in the last two decades by some CASA systems. Another problem associated with CASA motility was that it was based on video frames associated with either NTSC or PAL systems (restrictive in frame rates), and poor resolution cameras that were very restrictive. Furthermore, for reliable measurements and generating unbiased data, a concentration between 20 to 50×10^6^ sperm/mL has been recorded in some CASA studies [74–77].

### Kinematic parameters using computer-assisted sperm analysis

In addition to sperm motility and concentration, the CASA system can analyze different kinematic parameters including curvilinear velocity (VCL), straight-line velocity (VSL), average path velocity (VAP), linearity (LIN), straightness (STR), wobble (WOB), beat cross frequency (BCF), and amplitude of lateral head displacement (ALH). VCL analyzes the velocity of a sperm head on time-average basis along its actual curvilinear path (measured in in two dimensions). VSL measures the velocity of a sperm head on a time-average basis along the straight line between its first and last detected position. VAP measures the velocity of a sperm head along its average path on a time-average basis. This path is computed by smoothing the actual path according to algorithms in the CASA instrument, these algorithms vary among instruments. ALH measures the magnitude of the lateral displacement of a sperm head about its average path. This can be expressed as the maximum or an average of such displacement [69]. LIN estimates a curvilinear path reflecting the straightness of the sperm path (VSL/VCL×100 [%]); STR, reflecting the righteousness of motion (VSL/VAP×100 [%]); WOB, is the degree of oscillation of the actual path of the sperm head in its relationship with VAP (VAP/VCL×100 [%]). The progression of sperm in CASA also measures LIN, righteousness movement or STR, and Balancing or WOB, expressed as percentages.

ALH and BCF are measurements derived from VAP, where ALH is the amplitude of variations of the current path of the sperm head in its relationship with VAP, and BCF is the average rate at which the actual sperm trajectory crosses the VAP (a derivation of the true frequency of flagellar beat and frequency of rotation of the head). Although, CASA is a new technology and most of the progression of the sperm measurements are derived from the speed/velocity measurement, we did not find studies showing their correlation with fertility. Despite this, we recommend further studies should be performed on how these measurements can be utilized for assessing sperm fertility, unless there is no need to measure.

Rather than the PR, VAP is preferred to evaluate and predict the fertilizing potential of fresh or post-thawed bull semen [78]. The evaluation of sperm kinematic parameters with fertilizing capacity in bulls showed that all VCL, VSL, and VAP in post-thaw doses for AI were found to be correlated in achieving pregnancy [78–80]. From amongst the three kinematic parameters, VCL, VSL, VAP, VAP shows the highest correlation with fertility, and it may be the most useful sperm speed/velocity parameter, which can be relied upon for the estimation of sperm fertility [78].

### Flagellar and sperm tracking

The FAST method analyzes the movement of sperm by using the FAST program. FAST measures the flagellar beat frequency and the tangent and curvature of the flagellar wave to determine the arc-length (true length of the track). The analysis is usually conducted at higher frame rates, and tracks are represented quantitatively in three dimensions [71]. The FAST measures flagellar beat frequency, flagellar arc wave speed, flagellar arc wavelength (μm) (fAWL), Sperm flagellar length (μm), flagellar power dissipation tail length (fwatts), flagellar power dissipation first 30 μm (fwatts30), and potentially, also amplitude of flagellar displacement measured in TCS = track centroid speed (progressiveness) [62,71].

### Computer-assisted sperm analysis-three dimensions (3^D^) of sperm evaluation

The three dimensions (3^D^) system analyzes sperm tracks in three dimensions (X, Y, and Z axes); Z-axis is reconstructed as the sperm of most animal species are swimming in a spherical helix [62,81–83]. However, the 3^D^ method has the disadvantage that the Z-axis is presumed to be harmonic, but in most cases, it is not possible. In the 3^D^ patterns, there are clear differences, such as, in a bull sperm with a much higher speed and ALH, the helix diameter is almost twice that of boar sperm. A sperm of Saanen goat has similar VCL as that of a bull sperm, however, the BCF is an important factor here, showing small stepwise increases in the Z plane in Saanen goat sperm, in comparison to the larger Z-plane increases observed in bull sperm [62]. Although the 3^D^ method is useful in describing qualitative differences, it is not necessarily useful in describing quantitative differences. However, there is a need for further investigations to uncover the correlation between 3^D^ measurements and fertility indicators.

In combination, the findings of FAST and 3^D^ can be used for the advanced analysis of sperm-motility, such as measurements of sperm capacitation and hyperactivity, which was not possible with the conventional approach. This can be achieved by analyzing flagellar speed increase as well as energy expenditure (in Watts) over the first 30 μm length of the flagellum. There is a decrease in sperm flagellar beat frequency as it becomes capacitated and hyperactivated, resulting in tumbling sperm which shows swimming star spin patterns [62]. In general, CASA-based sperm analysis is one of the easiest, fastest, and reliable fertility assessments for semen evolution in individual animals, and it would be a method of choice for undertaking sperm evaluation comparative studies [84].

### The future of computer-assisted sperm analysis

The CASA analyzes sperm motility, which is directly correlated with fertility reported for bovine [85], equine [86], ovine [87], rabbit [88], and swine [89] sperm. What makes CASA essential is that it brought improvements in the quantitative analysis of the sperm quality in terms of accuracy and precision, as compared to conventional motility measurement methods [90–92]. It should be noted that there are various CASA system brands applicable for sperm evaluation, and standardization of the methods is of paramount importance to make objective measurements to precisely determine fertility [27,93].

The results of CASA should be defined in terms of the measurement conditions that are correlated with the outcome figures. In particular, the rate of image acquisition, time for tracking sample, smoothing algorithm, sperm concentration per sample, chamber’s type and depth, model and software version, microscope optic and magnification should be clearly defined to interpret CASA results to determine fertility in different species and conditions [94–97]. The setting of CASA measurements must be standardized for each species, else the instrument settings will affect the measurement results [27]. The results of CASA measurements can be interpreted into different parameters of sperm characteristics, including kinematic parameters, FAST, and 3^D^ sperm evaluation.

Over the decades, the CASA system has developed protocols to efficiently analyze the kinematic parameters of sperm in animals [98,99]. However, its use is not just limited to kinematic parameters, it can also analyze morphology, viability, DNA fragmentation, and acrosome reaction [100], which are considered vital for fertility determination. Computer-based semen analysis is striving to develop automatic microscopic systems by using artificial intelligence techniques (deep learning, machine learning, and computer vision).

Sperm morphology needs to be better elaborated by including the entire cell and especially understanding the tail characteristics by polychromatic staining in CASA. There seems to be a need for agreement in the selection of stains for use in conjunction with CASA for tail-characteristic studies. In future, developments could lead to no further need for staining and looking at the possibilities of more detailed analysis by using phase-contrast and Nomarski differential interference contrast optics. It is also expected that the CASA system will develop a new automatic analysis for new tests such as reactive oxygen species (ROS), mitochondrial assay, sperm maturity, sperm chromatin packaging, 3^D^ reconstruction, and tail analysis. This will further enhance our understanding of sperm functionality.

## PLASMA MEMBRANE INTEGRITY

The integrity of the plasma membrane is essential for proper sperm function and fertility, pertaining to its function to maintain homeostasis [101,102], protection against foreign agents, and interactions with other cells, including oocytes, and the epithelial lining of the female reproductive tract [103, 104]. Sperm fertility is dependent on the integrity of the plasma membrane, as it plays an important role in different physiological events such as capacitation, acrosomal reaction, and zona binding [105].

Plasma membrane integrity is usually analyzed for a viability test, as it is pivotal for communication with other cells and the environment [106]. Methods used for analyzing sperm plasma membrane are normally based on the enhanced permeability of damaged membrane [107]. These methods include the hypo-osmotic swelling (HOS) test [108,109], eosin–nigrosin staining [110,111], and use of fluorescent probes [112,113]. Fluorescent probes such as Hoechst or propidium-iodide (PI) are either used alone or in combination with other permeable fluorochromes such as carboxyfluorescein diacetate or SYBR [114–116]. Complementary techniques, including flow cytometry and fluorimetry, can be helpful in improving measurement precision, as large numbers of fluorochrome-stained sperm can be assessed in a short duration of time [117,118]. The recent development of the CASA technique has allowed the automated measurement of sperm plasma membrane integrity [119].

## VIABILITY AND ACROSOMAL STATUS

Sperm viability is a key factor for quality analysis and a prerequisite for success in fertilization, particularly in cases, such as AI, where low sperm numbers are used [120]. Eosin–nigrosin has been conventionally employed as a differential stain to assess the proportion of live and dead sperm. Eosin, being the cellular stain, stains the dead sperm with the damaged plasma membrane, whereas the live sperm will not attain any color and remain colorless and the nigrosin stains the background. In addition, several other stains have been widely used for sperm viability assessment, including fast–green and eosin, and opal–blue and eosin [121]. However, none of these methods has yielded consistently satisfactory results in comparison to that of eosin–nigrosin. Recently, sperm viability has been assessed using a combination of molecular probes, including SYBR-14 and PI [112]. After SYBR-14 and PI staining, live sperm percentage was determined by fluorescence microscopy, while flow cytometry was used to assess the uptake of stain. Live sperm are stained green with SYBR-14, whereas the dead sperm are stained red by PI.

Acrosomal integrity is one of the determining factors for fertility because, for successful fertilization, sperm must have an intact acrosome and must react on time when they reach the site of fertilization [122–124]. The ideal test used for the analysis of sperm acrosomal status should be precise, quick, applicable to samples with low sperm count, safe for sperm function, and must be able to differentiate normal from false acrosome reactions [125]. Previously, the acrosomal integrity of spermatozoa was assessed by fixing the sperm sample in 1% formal citrate solution [126]. Sperm with normal acrosomes have a sharp crescent-type appearance on the apical ridge. In addition, two classes of fluorescence probes were used for the assessment of acrosomal status. One probe involves the use of lectins and antibodies to detect intracellular acrosome-associated materials, whereas the other probes, used for live sperm, include chlortetracycline and antibodies against the exposed antigens [125]. The acrosomal status of spermatozoa can be determined by staining with fluorescein isothiocyanate-conjugated peanut agglutinin (FITC-PNA) [127] and fluorescein isothiocyanate-conjugated *Pisum sativum* agglutinin (FITC-PSA) [128]. FITC-PNA specifically binds to the sugar Galactosyl 61 β-1,3 N-acetyl galactosamine found in the acrosomal membranes [129], serving as a probe to visualize acrosomal integrity [130]. The limitation of using FITC-PSA is that it binds non-specifically with the sperm head and tail. However, cautions should be taken to distinguish true degenerative acrosome reactions and degenerative acrosome loss in FITC-PSA [128]. Later, the FITC-PNA methods were improved in 2019 [124].

## MITOCHONDRIAL INTEGRITY

Abnormal mitochondrial integrity or functionality in the supra structure or mitochondrial genome and low membrane potential of mitochondria or the change in level of oxygen consumption, are important for sperm function, affecting motility [131] and maturation [132] even protection of sperm against damage [133]. Mitochondrial function can be measured by rhodamine 123 (R123) fluorescence, when stain dye diffuses into the cell and accumulates in mitochondria [134,135] enabling mitochondrial damage detection, depending on the amount of fluorescence, and indicating the proportion of functioning mitochondria [120,136]. In addition to the R123 probe, JC-1 or Mitotracker dyes [137] can be used for the assessment of sperm mitochondria. The JC-1 dye cytometer is more sensitive and requires more careful preparation of staining conditions than other probes and must be used along with controls [138]. In bovine sperm, the DiOC_6_(3) probe can also be used, which has increased fluorescence compared to the JC-1 probe but lower specificity toward mitochondria [139].

## CHROMATIN INTEGRITY

The most common analytical tests used to assess sperm-DNA integrity include DNA fragmentation tests, measured by DNA fragmentation index (DFI) or sperm chromatin structure assay, and protamination, measured by sperm protamine deficiency assay [140]. These tests provide proof of sperm fertility by examining the level of DNA damage. DFI is found to be highly sensitive (79%) and specific (86%) for the detection of infertility in human sperm, and has higher accuracy than the conventional method of sperm evaluation [141]. DFI is one of the most common methods used to assess sperm DNA fragmentation. It can also be employed for fertility assessment of processed and cryopreserved sperm [142–144]. The higher the DFI percentage, the lower is the fertility in animals. In humans, a DFI of more than 30% indicates infertile sperm, which are unable to establish pregnancy [145].

The chromatin integrity of sperm is vital not only for fertility, but also for subsequent maintenance of gestation and offspring fitness [146]. The sperm chromatin assay is widely used for the assessment of chromatin status in sperm [146]. Chromatin integrity is essential for fertility, evidenced by the fact that higher DFI has been correlated with reduced fertility in boar [147], bull [148,149], and humans [150]. The sperm chromatin assay utilizes the metachromatic features of acridine orange, a DNA probe, and the principles of flow cytometry, which are correlated with classical sperm quality, whereby the intact chromatin are stained green and the damaged chromatin are stained orange [151]. In this assay, sperm containing greater red to green ratios exhibit more DNA denaturation than sperm exhibiting lower red to green ratios [150]. Moreover, male sperm DNA integrity is not only an indication of fertility, but it also affects embryonic development [145,146, 151].

Moreover, sperm chromatin integrity can be analyzed by detection of protamine-deficient sperm. During spermatogenesis, histone proteins are replaced by protamine to acquire a tightly packaged chromatin structure [152]. Sperm with protamine deficiency is an indication of loosely packed chromatin material [153]. Protamine deficiency can be directly detected using chromomycin A3 (CMA3) staining or indirectly by using an aniline blue (AB) staining procedure. CMA3 is a fluorochrome that competes with the protamine proteins for binding to the minor groove of DNA and emits green fluorescence, indicating protamine deficiency, thus reflecting abnormal chromatin [51]. AB stain binds to lysine-rich histone proteins and the sperm head is stained dark blue, indicating abnormal chromatin structure [152].

## REACTIVE OXYGEN SPECIES AND DNA DAMAGE MEASUREMENT

The ROS has both physiological and pathological roles, and an optimal amount is required for normal sperm function and fertilization [154]. Oxidate stress (OS) is an imbalance between the amount of ROS released and the ability of antioxidants to detoxify them. Higher ROS levels in semen can lead to OS, which is an important factor causing male infertility, reduced sperm motility, DNA damage, and increased risk of recurrent abortions and genetic diseases [155]. ROS-mediated peroxidation leads to DNA damage, base modification/strand breaks/chromatin cross-links, and also damages the plasma membrane, which accounts for defective sperm function observed in a high proportion of infertile patients, which results in cell apoptosis [156].

The ROS levels can be determined directly either by measuring the ROS levels or oxidation-reduction potential, or indirectly by measuring molecules, peroxidation, or DNA damage [157]. In humans, the ROS level can be measured using a direct method of luminal-mediated chemiluminescence [158], using the protocol [159]. The latest technique for measuring ROS levels directly, the MiOXSYS System, introduces a new strategy to detect OS by measuring the oxidation-reduction potential; a direct evaluation of the redox balance between ROS and antioxidants has shown promise as a diagnostic tool in the evaluation of male infertility [160,161]. Moreover, ROS levels can be measured indirectly by analyzing the expression levels of genes associated with DNA damage and mitochondrial ROS modulation. The methods developed include DNA fragmentation, chromatin integrity, sperm chromatin stability assay (SCSA modified), sperm chromatin dispersion, comet assay, transferase dUTP nick end labeling, and protamine evaluation in sperm chromatin assays, such as toluidine blue, CMA3, protamine expression, and cysteine radical evaluation [162].

## SPERM MATURATION AND CAPACITATION

Sperm maturation and capacitation are prerequisites for successful fertilization and are regulated by protein phosphorylation [163], the influx of calcium ions (Ca^2+^) to the sperm perinuclear and neck regions and the flagellum, and the generation of controlled amounts of ROS [106]. Capacitation mainly involves cytosolic pH changes and increasing Ca^2+^ levels, and Indo-1 acetoxymethylester (Indo-1 AM) has been used to measure intracellular Ca^2+^ in sperm with the help of flow cytometry [164] or fluo-3-acetomethoxy ester (Fluo-3 AM) probe [120,165,166].

## SPERM CYTOMETRIC ANALYSIS (FLOW CYTOMETRY)

Flow cytometry is a high-throughput technique that enables the analysis of thousands of sperm within seconds, capturing many physiological features of each sperm and can analyze sperm viability, acrosomal status, capacitation, mitochondrial status, apoptotic markers, OS markers, DNA damage, sperm count, and sperm size [138,167]. In general, the development of a flow cytometer is believed to revolutionize sperm analysis with further studies and understanding of the functions of sperm in wider animal species. Moreover, the advancement in semen evaluation by detecting the proteins in cytometry would give us a better insight into the structure-function relationship of a particular protein. Proteins with multiple post-translational modifications (PTMs) can be used to generate the complex interaction network involved in the physiological function of sperm. It can be speculated that crosstalk between different PTMs occurring either on the same or different proteins regulates protein stability and activity both in physiological and pathological states [168].

The viability and acrosomal status of sperm are vital in terms of determining sperm fertility. Cytometry can be used to assess the viability and acrosomal status of sperm. Viability of sperm can be assessed using PI and ethidium homodimer stains [169] or a combination of PI and SYBR-14 [170] or cyanine dye [171]. The acrosomal status is usually assessed by detecting targets/probes inside the acrosome, with the help of fluorochrome FITC test [172]. The disadvantage of the acrosomal reaction test is that it may result in false-negative results due to the disappearance of the binding sites by extensive acrosomal damage, either because of the heterogeneous labeling [173] or non-specific labeling [138] of the sperm.

## SEMEN PLASMA EVALUATION

Seminal plasma is a biological fluid contributed by the different accessory sex glands of male individuals. Seminal plasma is the supernatant recovered after centrifugation of liquefied semen, which constitutes >90% of semen [174]. It serves as a potential biological fluid for the discovery of novel fertility-related biomarkers and as clinical sample for noninvasive fertility diagnostics. Different components of seminal plasma play a wide range of physiological roles, including sperm nutrition and transportation, provision of an alkaline environment, liquefaction or coagulation of the ejaculate, and augmentation of immune response. The semen characteristics vary from species to species and within breeds of animals such as cattle [175], dog [176], and stallion [177]. The seminal plasma characteristics, which usually focus on measuring proteins as an indicator of fertility, affects the OS [178], function, survival, and transport of sperm in the female reproductive tract [179]. In humans, the plasma protein has essential role in fertility, and it is also involved in the inflammatory and immune response to sperm during transit through the reproductive female tract in animals [180] and is associated with the fertility of bulls [181].

As with the proteomic analysis of blood plasma, owing to the wide dynamic range of protein concentrations, identification of low-abundance proteins in seminal plasma by mass spectrometry is challenging. Particular obstacles are the high-abundance proteins expressed in the seminal vesicles and prostate [174]. Polyacrylamide denaturation test (gel electrophoresis) is used for the detection of protein metabolites and has proved to be helpful for the identification of certain proteins that can serve as biomarkers of fertility. In stallions, two proteins were found to be positively correlated with fertility, and another two, with infertility [182]. In Holstein-Friesian bull, the proteins associated with fertility in seminal plasma represent the major protein fraction of bovine seminal plasma [183]. The same seminal plasma protein is found to be associated with fertility in different species, such as horse, goat, and bison [184].

### Seminal plasma evaluation by liquid chromatography

Liquid chromatography analyzes the presence of specific metabolites associated with sperm fertility [185]. Metabolites can be detected in seminal plasma or serum as an indicator of fertility. Metabolites are products of biochemical pathways that are considered more representative of phenotypic traits and hence, are more reliable [186]. The metabolites are outcomes resulting from transcriptional and translational changes that are significantly associated with one or more already established individual sperm quality parameters (motility, concentration, total count, and morphology) [185].

Assessing metabolites could be helpful in estimating a bull's fertility even without manipulating sperm [186]. This technique is superior to genomic and proteomic analysis, because in genomic techniques, results are affected by post-transcriptional and post-translational changes, and leads to variation in the sperm phenotypic characteristics [186]. For instance, in Holstein bull’s seminal fluid, fructose is the most abundant metabolite, followed by citric acid, lactic acid, urea, and phosphoric acid, whereas, androstenedione, 4-ketoglucose, D-xylofuranose, 2-oxoglutaric acid, and erythronic acid represent the least predominant metabolites [187]. For separation between high-and low-fertility groups, 2-oxoglutaric acid and fructose are taken as indicators of high fertility in bulls [187]. In humans, plasma proteins vary according to fertility, with lactate, citrate, glycerylphosphorylcholine, and glycerylphosphorylethanolamine levels being higher in the seminal fluid in the infertile as compared to the fertile [188]. In addition, epididymal function and prostate function are correlated with the presence of citric acid, α-glutamyl transferase, and acid phosphatase in seminal plasma [189]. The presence of leptin in serum is also correlated with proper sperm morphology [190]. Recently, in China, the presence of higher monobutyl phthalate (toxic substance) in human semen has been found to be associated with decreased sperm concentration and total count, whereas di-(2-ethylhexyl)-phthalate metabolites were associated with an increased percentage of sperm head abnormalities [191–193].

### Semen plasma evaluation by spectrophotometry

The seminal plasma examination in bulls can be conducted using a proton nuclear magnetic resonance spectrometer. This method showed that citrate and isoleucine were low, whereas tryptamine/taurine and leucine were greater in high fertility bulls than in low-fertility bulls, which can be identified by peaks in seminal plasma [186]. In serum, the identifiable level can be used for the diagnosis and determination of fertility in bulls, whereby, isoleucine and asparagine were lower, and glycogen and citrulline were significantly greater in high fertility bulls [186]. Semen evaluation by spectrometry is highly sensitive and can measure the phenotypic sperm quality characteristics, but it requires well-equipped laboratories, limiting its wider application [194]. We recommend a comparative evaluation of the seminal plasma from different species of animals, which may be used in research and for defining the fertility in animals, where AI is used.

## SPERM TRANSCRIPTOME ASSAY (GENE EXPRESSION)

Gene expression pattern of the sperm, in terms of defined fertility indicators, was found to be an important indicator of fertility. The transcriptomic profiling of human sperm assumes significance as it carries information about spermatogenesis, sperm function, and paternal roles in post-fertilization events [25]. In bovine studies on sperm transcripts revealed thousands of gene transcripts associated with the events of spermatogenesis [25,195].

In bovines, the genes related to spermatogenesis are protamine 1 (*PRM1*), insulin like growth factor 1 (*IGF1*), bone morphogenetic protein 2 (*BMP2*); sperm function are, testis specific serine kinase 6 (*TSSK6*), cysteine-rich secretory protein (*CRISP*), heat shock transcription factor Y-linked (*HSFY2*); fertility are, ubiquitin-conjugating enzyme E2 D3 (*UBE2D3*), integrin-β, leucine decarboxylase 1 (*LDC-1*); and embryonic development are, miR34c-5p, B-cell lymphoma-2-like protein 11 (*BCL2L11*), breast cancer type 1 (*BRCA1*). The most abundant translated bovine transcripts are binder of sperm 3 (*BSP3*) and spermatogenesis associated 18 (*SPATA18*), which are involved in the regulation of germ cell development and the maintenance of chromatin integrity during spermatogenesis, respectively [25]. The advantages of examining transcriptomic gene quality are its ability to predict semen quality significance as it carries information about spermatogenesis, sperm function, and paternal roles in post-fertilization events [25].

Improved gene expression test, in lieu of the conventional and CASA assessments, is associated with phenotypic traits of developing embryos and offspring, and can identify idiopathic infertile males [196,197]. For example, the embryogenesis-associated transcripts were highly abundant, and transcripts such as *BCL2L11* and *BRCA1* existed as intact and full-length molecules in sperm. The molecular functions of the highly abundant sperm transcripts are associated with the structural components of ribosomes, whereas the less abundant transcripts are associated with ion transporter activity, and these transcripts are constituents of cytosolic ribosomes and main axons [198]. Furthermore, placenta-associated genes, such as thrombospondin type-1 domain-containing protein 4 (*THSD4*), pregnancy-associated glycoprotein 5 (*PAG5*), *PAG7*, and *PAG10*, were abundantly transcribed in sperm, suggesting their possible role in implantation and placental development [198]. The proper organization of the centrosome of sperm, in the function of the sperm transcriptome tektin-1 (*TEKT1*) is essential for oocyte genome activation leading to successful fertilization, and any defects in this process might lead to male infertility [199]. *MAP7*, *PTK2*, *PLK1S1*, microtubule associated protein 7 (*MYH9*), and protein kinase C zeta type (*PRKCZ*) are essential for spermatogenesis and sperm function [25].

Polymerase chain reaction (PCR)-based techniques are best due to their more quantitative nature, but their major limitation is the need for prior knowledge of the gene sequences to be assessed [25,200]. Most studies indicate that sperm RNA levels can be used to study the past events of spermatogenesis, sperm function, and successful fertilization in cattle and pig [198,201]. Subtle changes in sperm transcripts and proteins are not assessed as part of the standard semen evaluation procedure. Through fertilization, these seemingly normal spermatozoa may yet be detrimental to the post-fertilization development where the spermatozoal RNAs play a crucial role [25].

Cryopreserved semen can be assessed for its general characteristics by CASA, but to assess the phenotypic normality of sperm, we propose that further gene expression assessment be done, particularly if the genetic materials in the same cycle are going to be used in wider scales [202–204]. Gene expression, in terms of the composition of sperm RNA can be analyzed using hybridization, quantitative (real-time) PCR, microarray, and sequencing techniques, which reflect the level of sperm maturation, function, quality, and successful outcome of fertilization and zygote development [25,205, 206].

## CONCLUSIVE REMARKS

Semen analysis remains the only choice for accurate prediction of fertility in males. The advancement in semen evaluation methods still requires refinement, species-specific standards, and quality control. The prediction of fertility in interspecies and/or intraspecies by a single analytical method is not yet possible due to the complexity of different tests. Various methods can be used to estimate sperm quality parameters, even though few techniques require shorter time. Various techniques have their own pitfalls and the possibility of variability; a combination of tests is therefore required to achieve high correlations of test results with fertility. The CASA system mainly assesses motion characteristics of sperm and despite the addition of morphology and concentration parameters, it is impossible to evaluate all sperm quality parameters for species with relatively small head size at the same time. Moreover, the major problem with the automatic CASA system is the inability to differentiate sperm from cells, cytoplasmic droplets, and cellular debris of similar size.

The flow cytometry method shows high sensitivity and reproducibility for DNA analysis but requires expensive equipment and proper training. Similarly, seminal plasma spectrometry reveals the results of the actual metabolism and is more accurate in fresh semen, but it was not well determined for most species. Gene expression demonstrated a marked improvement in fertility prediction, enabling the measurement of DNA fragmentation and the normality of acrosome, particularly in post-thaw sperm. However, the requirement of molecular laboratories, capital inputs, and more skilled personnel limits its use for research purposes only. We recommend the use of seminal plasma metabolite analysis for different animal species as the last level of fertility assessment.

Finally, we can conclude that the conventional semen analysis must undergo quality control measures, and reference values should be followed for clinical interpretation of results. Similarly, the primary basis of semen assessment requires constant visual supervision, calibration by trained personnel, and objective evaluation. Therefore, it is recommended that novel tests should be incorporated into the clinical andrology.
